# Status of the neutron time-of-flight single-crystal diffraction data-processing software *STARGazer*

**DOI:** 10.1107/S2059798318012081

**Published:** 2018-10-29

**Authors:** Naomine Yano, Taro Yamada, Takaaki Hosoya, Takashi Ohhara, Ichiro Tanaka, Nobuo Niimura, Katsuhiro Kusaka

**Affiliations:** a Frontier Research Center for Applied Atomic Sciences, Ibaraki University, 162-1 Shirakata, Tokai, Ibaraki 319-1106, Japan; b College of Engineering, Ibaraki University, 4-12-1 Nakanarusawa, Hitachi, Ibaraki 316-8511, Japan; c Neutron Science Section, J-PARC Center, Japan Atomic Energy Agency, 2-4 Shirakata-Shirane, Tokai, Ibaraki 319-1195, Japan

**Keywords:** neutron time-of-flight single-crystal diffraction data, data-processing software, iBIX, *STARGazer*

## Abstract

The *STARGazer* data-processing software is used for neutron time-of-flight (TOF) single-crystal diffraction data collected using the IBARAKI Biological Crystal Diffractometer (iBIX) at the Japan Proton Accelerator Research Complex (J-PARC). This software creates *hkl* intensity data from three-dimensional (*x*, *y*, TOF) diffraction data. *STARGazer* is composed of a data-processing component and a data-visualization component. The former is used to calculate the *hkl* intensity data. The latter displays the three-dimensional diffraction data with searched or predicted peak positions and is used to determine and confirm integration regions. *STARGazer* has been developed to make it easier to use and to obtain more accurate intensity data. For example, a profile-fitting method for peak integration was developed and the data statistics were improved. *STARGazer* and its manual, containing installation and data-processing components, have been prepared and provided to iBIX users. This article describes the status of the *STARGazer* data-processing software and its data-processing algorithms.

## Abstract 2

In this article, the status of the *STARGazer* data-processing software and its data-processing algorithms are described in detail. The *STARGazer* data-processing software is used for neutron time-of-flight single-crystal diffraction data collected using the IBARAKI Biological Crystal Diffractometer.

## 1. Introduction   

Hydrogen is one of the main atoms that proteins are composed of, and it plays an important role in protein function and structure. X-rays are often used to solve protein structures, but it is difficult to determine the position of H atoms without ultrahigh resolution. Additionally, because protons have no electrons, we cannot theoretically determine proton positions using X-rays. Neutron protein crystallography (NPC) is used to determine H-atom and proton positions in proteins, and plays very important roles in understanding physiological functions and reaction mechanisms (Niimura *et al.*, 2016 ▸; O’Dell *et al.*, 2016 ▸). Several neutron instruments for protein crystallography have been installed at research reactors that use monochromatic neutrons or relatively narrow bands of neutrons (with wavelengths from 3 to 4 Å) emitted by monochromators or multilayer band-pass filters, respectively. BIX-3 (Tanaka *et al.*, 2002 ▸) and BIX-4 (Kurihara *et al.*, 2004 ▸) at Japan Research Reactor No. 3 (JRR-3) and BIODIFF at the Research Neutron Source Heinz Maier-Leibnitz (FRM-II) are monochromator-type diffractometers. LADI-III (Blakeley *et al.*, 2010 ▸) at the Institute Laue–Langevin (ILL) and IMAGINE (Munshi *et al.*, 2011 ▸) at HFIR (High Flux Isotope Reactor) are quasi-Laue-type diffractometers. The neutron time-of-flight (TOF) method uses pulsed neutrons with continuous wavelengths generated at accelerator-driven high-intensity spallation neutron sources. Because the velocity of a neutron depends on its wavelength, the flight times of neutrons from their sources (the moderator) through the sample to the detectors vary. Thus, we can calculate the neutron wavelength by measuring the flight times, and separate diffraction peaks at the same detector pixel and different wavelengths using fixed time-resolved detectors. In this manner, the TOF method can save data-collection time compared with the monochromatic or quasi-Laue methods (Niimura & Podjarny, 2011 ▸). The IBARAKI Biological Crystal Diffractometer (iBIX; Tanaka *et al.*, 2010 ▸; Kusaka *et al.*, 2013 ▸) at the Japan Proton Accelerator Research Complex (J-PARC; Ikeda, 2009 ▸), the Protein Crystallography Station (PCS; Chen & Unkefer, 2017 ▸) at Los Alamos Neutron Science Center (LANSCE; Cooper, 2006 ▸) and the Macromolecular Neutron Diffractometer (MaNDi; Coates *et al.*, 2015 ▸) at the Spallation Neutron Source (SNS; Mason *et al.*, 2000 ▸) are TOF neutron diffracto­meters for NPC. Additionally, the NMX macromolecular diffractometer at the European Spallation Source (ESS; Hall-Wilton & Theroine, 2014 ▸) is under construction and will soon be operational.

**Figure 1:**
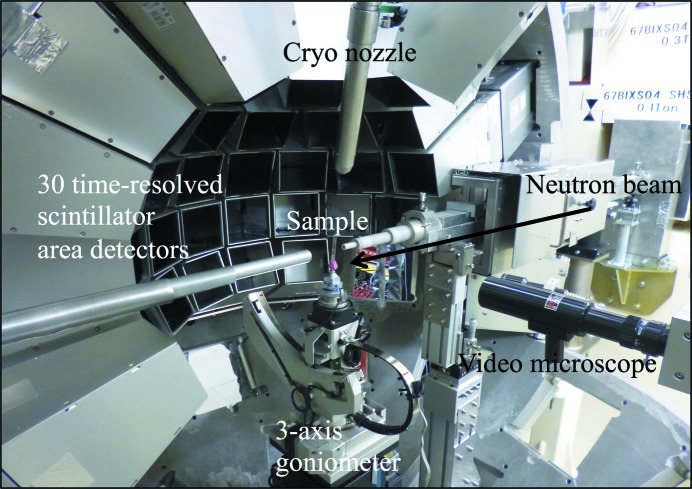
Internal view of iBIX, equipped with 30 two-dimensional time-resolved scintillator area detectors, a three-axis goniometer with ω, χ and φ axes, and a cryonozzle. The active area of each detector is 133 × 133 mm and the distance from the sample to each detector face center is 490 mm. The cryonozzle corresponds to the nitrogen and helium cold gas streams for low-temperature measurements.

iBIX, which is installed on beamline BL03 at the Materials and Life Science Experimental Facility (MLF; Nakajima *et al.*, 2017 ▸) at J-PARC, is a neutron TOF single-crystal diffracto­meter that is mainly utilized for elucidating the hydrogen, protonation and hydration structures of biological macromolecules in various life processes (Yokoyama *et al.*, 2012 ▸, 2015 ▸; Ogo *et al.*, 2013 ▸; Unno *et al.*, 2015 ▸; Nakamura *et al.*, 2015 ▸). It possesses 30 globally placed time-resolved scintillator area detectors (Hosoya *et al.*, 2009 ▸), each with active areas of 133 × 133 mm (256 × 256 pixels), a three-axis goniometer with ω, χ and φ axes, and a cryonozzle to inject nitrogen and helium cold gas streams for low-temperature measurements (Fig. 1 ▸). iBIX is installed on the H_2_-coupled moderator (CM) beamline and the flight-path lengths from the CM to the sample and from the sample to the detector face centers are 40 m and 490 mm, respectively (Kusaka *et al.*, 2013 ▸). As iBIX is installed on the CM beamline, it produces a significant broadening of the neutron pulse, leading to an asymmetrically shaped neutron pulse in the direction of the TOF axis. However, the intensities of pulsed neutrons from the CM are stronger than those from H_2_-decoupled moderators or poisoned decoupled moderators (Maekawa *et al.*, 2010 ▸). While oscillation diffraction data are recorded using the monochromatic method, diffraction data in the nonmoving state are recorded at various crystal orientations in the TOF method.

In 1972, the first TOF neutron diffraction experimental facility in the world was constructed at the Tohoku Electron Linac, which included a unique data-acquisition and reduction system. In 1980, a new spallation neutron facility, KENS (KEK Neutron Source), was built and housed a similar successful data-acquisition and reduction system. However, the ISIS *Genie* system (Campbell *et al.*, 2002 ▸) was later introduced at KENS because its visualization components were well established. Gradually, several next-generation spallation neutron sources were constructed all over the world. Because each TOF neutron diffractometer differs in terms of the number of detectors, the detector arrangement, the detector active-area size, the installed moderator *etc.*, TOF NPC diffraction data-processing software was developed independently at each diffractometer facility. *STARGazer* (Ohhara *et al.*, 2009 ▸), d*TREK modified for wavelength-resolved Laue neutron crystallography (Langan & Greene, 2004 ▸) and *Mantid* (Arnold *et al.*, 2014 ▸) are used at iBIX, PCS and MaNDi, respectively.

When we started to develop the data-acquisition and reduction system for iBIX, three software systems, namely d*TREK modified for wavelength-resolved Laue neutron crystallography, *ISAW* (Mikkelson *et al.*, 2005 ▸) developed at the IPNS (the Intense Pulsed Neutron Source at Argonne National Laboratory) and software for the single-crystal neutron diffractometer SXD at ISIS, were already available for the reduction of data from TOF single-crystal diffracto­meters. Among these, only d*TREK could be used for TOF NPC. The data-reduction software used at SXD included many useful functions for data reduction. However, because this was not open-source software, it was difficult to use as the basic component for data reduction of TOF data at iBIX. The modified d*TREK was also not open-source software. The integration algorithm of modified d*TREK is based on integration from the *X*–*Y* two-dimensional map for each TOF bin. Because we attempt to obtain the integrated intensity using the profile-fitting method in the TOF direction and to apply the profile-fitting method to the peak separation of the overlapped reflections of larger unit-cell crystals, this algorithm is not appropriate for data reduction at iBIX. For these reasons, we decided to develop data-reduction software for iBIX TOF diffraction data sets based on the *ISAW* algorithm, which is an open-source program in which the integration algorithm calculates the integrated intensity directly from three-dimensional histogram data. However, the *ISAW* algorithms used for peak search and determination of the **UB** matrix were so simple that the algorithms used in several components of *ISAW* were not sufficient to carry out the data reduction of diffraction data from protein single crystals measured by iBIX. Therefore, we had to develop an original function for the data-reduction process in *STARGazer*. For example, *STARGazer* should process data from protein single crystals with weaker and broader peak intensities than those of organic or inorganic compounds. In addition, we need to develop visualization software for TOF diffraction data to check the data quality and determine several parameters for data reduction. *X*–*Y*, *X*–TOF and *Y*–TOF two-dimensional slice maps and a one-dimensional TOF profile should be visualized from the three-dimensional histogram data. Components to calculate the data statistics, such as the number of observed reflections, the number of independent reflections, the averaged multiplicity, the completeness (%), *R*_merge_, *R*_p.i.m._ and *I*/σ(*I*), have also been developed to estimate the degree of coincidence of equivalent reflection intensities and the data quality, especially for the determination of the resolution limit. *STARGazer* was developed using the C++ and Python programming languages by rewriting the *ISAW* algorithms in C++ and adding algorithms for protein single crystals. *STARGazer* mainly consists of two parts: data processing and data visualization. The former is used to calculate *hkl* intensity data. The latter displays the three-dimensional diffraction data with searched or predicted peak positions and is used to determine and confirm integration regions. We have developed *STARGazer* to improve the quality of intensity data and to make it easier to use. The *STARGazer* manual consists of an installation part and a data-processing part, and has been prepared and offered to iBIX users.

In this article, the status of the *STARGazer* data-processing software and its data-processing algorithms are described.

## 2. Overview of *STARGazer*   

### 2.1. Platform   

*STARGazer* can be used on Linux, Mac and Windows by using a free and open-source hypervisor called VirtualBox (https://www.virtualbox.org). Users first download and install VirtualBox on their PC and boot CentOS 6.4, which installs *STARGazer*, on VirtualBox. Users can then begin data processing. The boot file and installation manual are distributed to iBIX users free of charge. *STARGazer* source code is included in the boot file. When new versions of *STARGazer* are released, the boot file and manual are also revised. Users who require information regarding data processing can attend a workshop at any time, and any questions are accepted by e-mail.

### 2.2. Data-processing component   

**Figure 2:**
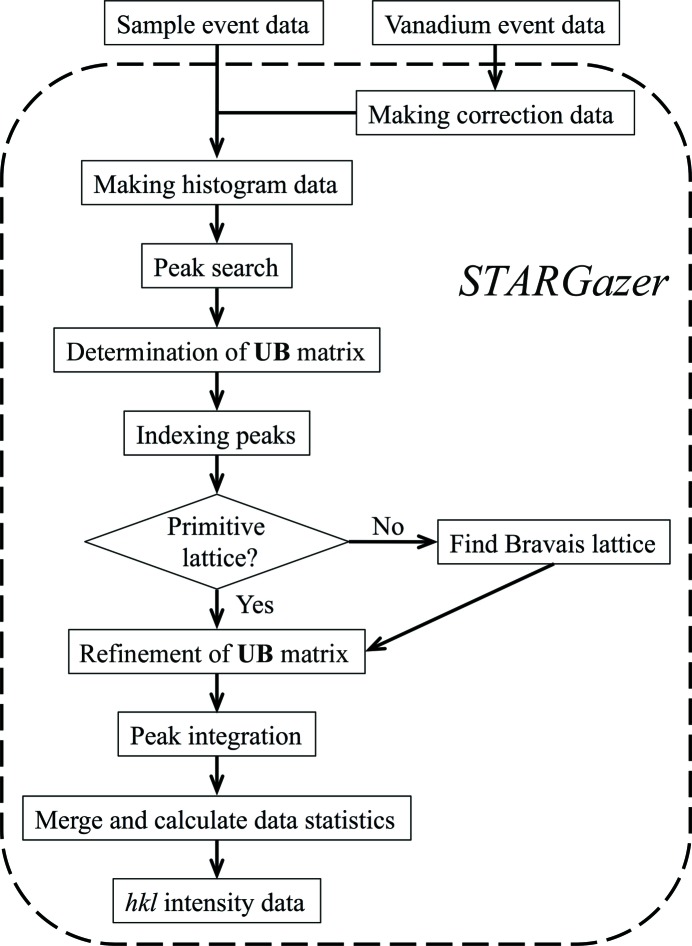
Flowchart of data processing in *STARGazer*.

**Figure 3:**
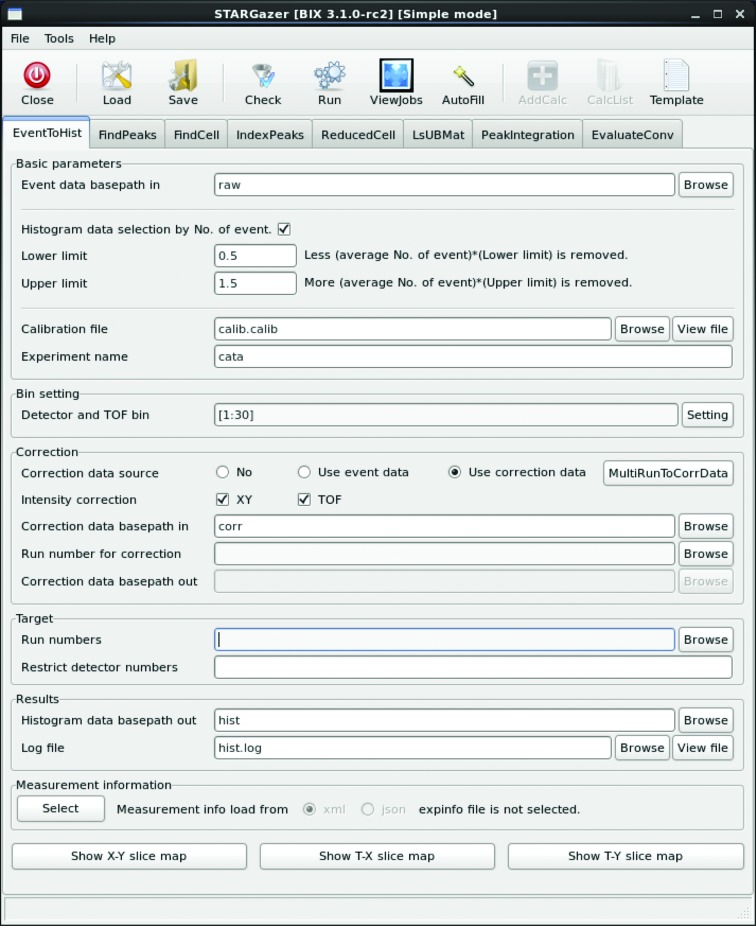
Graphical user interface in the EventToHist component. Input and output directory names and file names can be set with the ‘Browse’ button. Input and output text files can be viewed with the ‘View file’ button. Histogram data can be viewed from the ‘Show X-Y slice map’, ‘Show T-X slice map’ and ‘Show T-Y slice map’ buttons.

The data-processing component creates *hkl* intensity data from neutron diffraction data. It is composed of eight components, EventToHist, FindPeaks, FindCell, IndexPeaks, ReducedCell, LsUBMat, PeakIntegration and EvaluateConv, which are described below. A flowchart of the data-reduction process is shown in Fig. 2 ▸. The data-processing component includes the graphical user interface (Fig. 3 ▸) and users can carry out data reduction easily. Because the input and output file sizes are large, it is recommended that users save these files to external hard disk drives. The recommended hard disk drive specifications are 1.2 TB available storage, USB 3.0 and 7200 rev min^−1^ to improve file reading and writing times.

#### 2.2.1. EventToHist   

The raw neutron diffraction data collected at iBIX are event data that record the spatial detector position at *x*, *y* and the TOF of each detected neutron. Because the neutron count in most *x*, *y* and TOF positions is zero, event data can save on diffraction data size. For data processing, it is suitable to convert event data to a histogram that records the neutron count at each *x*, *y* and TOF. On the other hand, synchrotron X-ray diffraction data are histogram data, so this procedure is not necessary for X-ray data-processing software. The pixel counts are binned in the TOF direction to form channel *t* and histogram data are dealt with as *x*, *y* and *t* data. For example, as iBIX can record in a 40 000 µs range in the TOF direction, we can create a 1000-channel histogram in which each channel has a bin width of 40 µs.

Because the neutron time-of-flight method uses pulsed neutrons with continuous wavelengths and iBIX possesses 30 globally placed detectors, when creating sample crystal histogram data the variance in the detection efficiency of pixels within one detector, the difference in neutron beam intensities by wavelength and the difference in detection efficiency by wavelength are corrected using correction data. On the other hand, these corrections are not necessary for synchrotron X-ray data because single-wavelength X-rays are used and typically only one detector is present. The event data from incoherent scattering of a vanadium sphere 4.8 mm in diameter by a 5 mm diameter incident neutron beam are used as the correction data. Two vanadium event data are collected using wavelength ranges of 0.06–3.94 and 2.88–6.76 Å. The two data are scaled by the number of neutrons irradiating the vanadium sphere, and are combined according to sample-data wavelength range and connecting wavelength. Additionally, the difference in the total number of neutrons irradiating the sample crystal at each sample orientation is corrected. Considering one detector, the corrected pixel counts cnt′(*x*, *y*, *t*, *i*) of the *i*th sample-crystal orientation at detector position *x*, *y* and *t* is 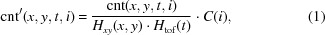
where cnt(*x*, *y*, *t*, *i*) is the number of counted neutrons of the *i*th sample-crystal orientation before correction at *x*, *y* and *t*. The other terms are described below.

**Figure 4:**
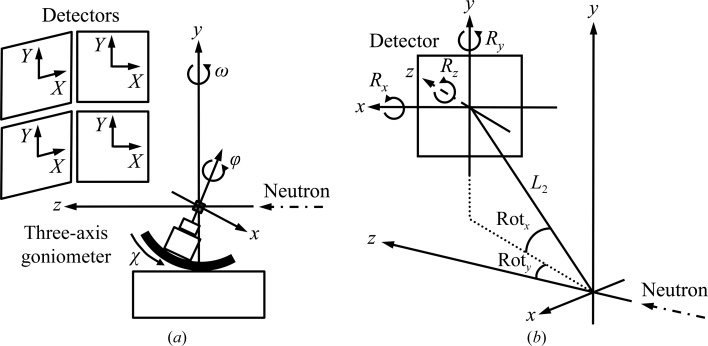
(*a*) Definition of the detector coordinate system (*X*, *Y*), diffractometer coordinate system (*x*, *y*, *z*) and direction of positive rotation ω, χ and φ for the goniometer spindle axis. (*b*) Detector parameters. Rot_*x*_ and Rot_*y*_ represent the detector position angles. *R_x_*, *R_y_* and *R_z_* are the detector mis-setting angles. *L*_2_ is the distance between the crystal and the detector face center. The *x*- and *z*-­axis directions of the detector coordinate system are reversed to calculate the peak position in the diffractometer coordinate system.

iBIX detector positions are designated as 256 × 256 pixels and each pixel has a variance in detection efficiency. To correct this variance, a correction factor *H_xy_*(*x*, *y*) is calculated using correction data, 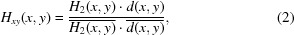
where *H*_2_(*x*, *y*) is the summed counts of the two-dimensional correction data histogram between sample minimum and maximum TOF in the TOF direction at a specific *x*, *y* position, 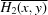 is the average of all pixels *H*_2_(*x*, *y*), *d*(*x*, *y*) is the square of the distances from the sample crystal to each detector pixel position (*x*, *y*) and is used to correct intensity attenuation using the difference in distance from the sample crystal to each detector pixel position, and 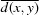 is the average of all pixels *d*(*x*, *y*). It is assumed that neutron intensity is in inverse proportion to the square of the distance from the sample crystal to each detector pixel position. *d*(*x*, *y*) is calculated from the pixel position considering the detector mis-setting angles *R_x_*, *R_y_* and *R_z_*. Because the directions of the *x* and *z* axes of the detector coordinate system are opposite to those of the diffractometer coordinate system (Fig. 4 ▸ *a*), the *x* and *z* axes are reversed and the coordinates of the pixel position on the detector are transformed as
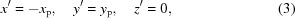
where *x*_p_ and *y*_p_ are the pixel positions on the detector. The coordinates of the pixel position considering the detector mis-setting angles are calculated as
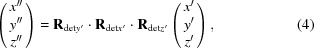
where **R**_det*x*′_, **R**_det*y*′_ and **R**_det*z*′_ are 3 × 3 matrices used to rotate the detector face around the *x*, *y* and *z* axes, respectively (Fig. 4 ▸ *b*). **R**_det*x*′_, **R**_det*y*′_ and **R**_det*z*′_ are defined as
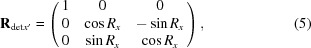


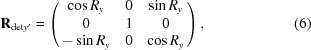


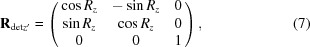
where *R*_x_, *R*_y_ and *R*_z_ are the detector mis-setting angles around the *x*, *y* and *z* axes, respectively. *d*(*x*, *y*) is given by
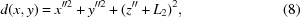
where *L*_2_ is the distance between the detector face center and the sample crystal center.

To correct for the difference in neutron beam intensities and the detection efficiency of pixels by wavelength, a correction factor *H*_tof_(*t*) is calculated using the correction data as follows. When the TOF channel bin settings of the correction and sample data are equal, then
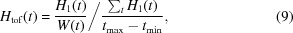
where *H*_1_(*t*) is the one-dimensional correction data histogram summed counts in all *x* and *y* at a certain *t* channel, *W*(*t*) is the bin width of channel *t*, and *t*_min_ and *t*_max_ are the minimum and maximum TOF values, respectively, of the sample crystal data.

When measuring neutron diffraction data at various crystal orientations, the numbers of neutron pulses proportional to the number of neutrons irradiating the sample crystal are normally identical at each crystal orientation. However, because proton accelerator power is not always constant, the total number of neutrons irradiating the sample at each crystal orientation is different. A correction factor *C*(*i*) is applied, 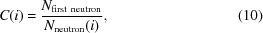
where *N*_first neutron_ and *N*_neutron_(*i*) are the total numbers of neutrons irradiating the sample at the first and *i*th crystal orientations, respectively.

When *H_xy_*(*x*, *y*)·*H*_tof_(*t*) in (1) is lower than 10^−5^, the corrected pixel counts are regarded as zero. After data correction, histogram data are output and can be displayed in the data-visualization component (see §[Sec sec2.3]2.3).

#### 2.2.2. FindPeaks   

**Figure 5:**
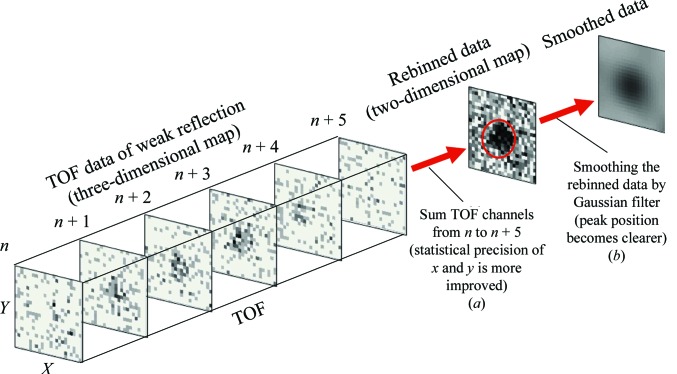
Determination process of the *x* and *y* positions of a peak by the rebinning and smoothing method. The TOF position is determined by a similar process.

This component searches the peak position of the reflections from the three-dimensional histogram data. The main target of measurement by iBIX is a protein single crystal. Because neutron beam intensity is considerably lower than synchrotron X-ray beam intensity, and the quality of protein single crystals is lower than those of organic or inorganic compounds, the peak intensities of neutron diffraction from protein single crystals are weaker and broader. Thus, it is difficult to search for the peak positions of the reflections using simple peak-search algorithms. To address these problems, a rebinning and smoothing method was developed for and implemented in the FindPeaks component. Because the neutron beam intensity is weak, the three-dimensional peaks of the reflections have a few counts at each pixel, and it is difficult to determine whether these are real peaks. Tens of TOF channels are summed to increase the count of each *x*, *y* pixel and rebinned histogram data are calculated (Fig. 5 ▸ *a*). Rebinned histogram data are smoothed in the *x* and *y* directions by replacing the count at each pixel point with a weighted average of the count values within the surrounding region (Fig. 5 ▸ *b*). Pixels with counts larger than the threshold, and the largest of the surrounding 3 × 3 pixels, are selected as the positions of the peak candidates. After determination of the *x* and *y* positions of the peak candidates using the rebinned histogram data, the TOF positions of the peak candidates are determined using the smoothed TOF profile of the reflection obtained from the original histogram data. Bins with counts larger than the threshold and the largest of the surrounding three bins are selected as the positions of peak candidates. Because the positions of peak candidates can be densely populated, a candidate peak with a maximum count in one region is selected as a peak.

**Figure 6:**
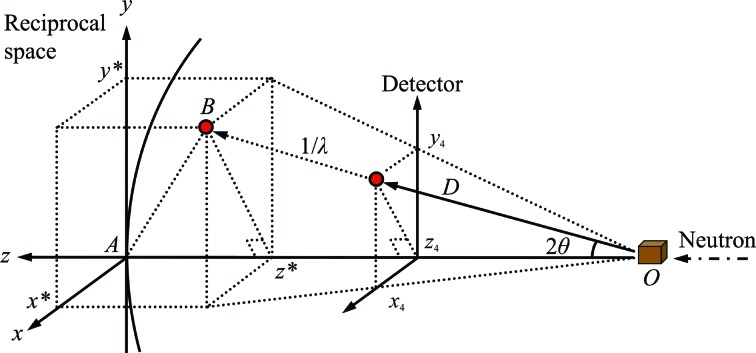
Diffractometer and reciprocal-space coordinate systems. The neutron beam is projected from the right-hand side and diffracted neutrons from the crystal at *O* create a scattering angle of 2θ with the incident beam direction. The reciprocal-lattice point is shown as a red sphere at *B* and is located on the Ewald sphere surface. Hence, *OA* = *OB* = 1/λ. z* = −(*OA* − *OB*cos2θ) = − {(1/λ) − [(1/λ)cos2θ]} = (1/λ)[(*z*_4_/*D*) − 1]. We can also state that x* = *x*_4_/*D*λ and y* = *y*_4_/*D*λ.

After the peak search, the positions of the peaks are corrected. The centers of gravity in the *x* and *y* directions are calculated from the background-subtracted neutron count of the searched peak and around the peak position, respectively. The peak positions at *x* and *y* are set to this position. When pulsed neutrons are generated, the pulse intensity begins to increase at TOF = 0 µs and reaches a maximum intensity after several to hundreds of microseconds. This time lag is named the TOF offset, and the searched peak positions at the TOF minus the TOF offset are used as corrected peak positions. Because the TOF offset differs with neutron wavelength, TOF offsets are calculated for each peak based on the wavelength. The neutron wavelengths λ of each peak are calculated from the peak positions at the TOF and de Broglie’s equation,
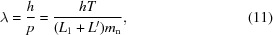
where *h* is the Planck constant, *p* is the momentum, *T* is the TOF of the detected neutron, *L*_1_ is the distance from the moderator to the sample (40 m for iBIX), *L*′ is the distance from the sample to the detector pixel where the neutron is detected, and *m*_n_ is the mass of a neutron. *L*′ is calculated from (8). Additionally, the reciprocal-lattice coordinates of each peak are calculated from the peak positions on the detector, the detector mis-setting angles *R_x_*, *R_y_* and *R*_z_, the distance from the sample to the detector face center, the detector position angles Rot_*x*_ and Rot_*y*_ and the wavelength of the neutron. The peak positions on the diffractometer coordinate system are calculated from the peak positions on the detector coordinate system. Because the directions of the *x* and *z* axes of the detector-coordinate system are opposite to those of the diffractometer coordinate system (Fig. 4 ▸ *a*), the *x* and *z* axes are reversed, and the transformed coordinate of the peak position on the detector is
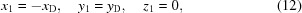
where *x*_D_ and *y*_D_ are the peak positions on the detector. The coordinates of the peak position considering the detector mis-setting angles are calculated as
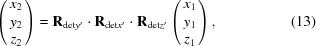
where **R**_det*x*′_, **R**_det*y*′_ and **R**_det*z*′_ are the same as in (5)–(7) [Disp-formula fd5] [Disp-formula fd6] [Disp-formula fd7](Fig. 4 ▸ *b*). Because the detector face centers are far from the sample center, *L*_2_, the distance from the detector face center to the sample center, is added to the *z* coordinate.
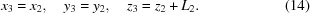
Additionally, the peak positions are rotated by the detector position angles, 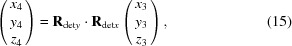
where **R**_det*x*_ and **R**_det*y*_ represent the 3 × 3 matrices to rotate the peak position around the *x* and *y* axes on the diffracto­meter coordinate system, respectively. **R**_det*x*_ and **R**_det*y*_ are given by
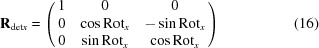
and
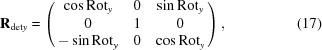
where Rot_*x*_ and Rot_*y*_ are the detector position angles around the *x* and *y* axes, respectively (Fig. 4 ▸ *b*). The reciprocal-lattice coordinates Q* of each peak are calculated from the peak position on the diffractometer coordinate system (Fig. 6 ▸) as
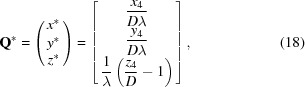
where *D* is the distance from the peak position to the sample,
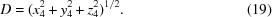



#### 2.2.3. FindCell   

The **UB** matrix is determined after the peak search; this is a 3 × 3 matrix that represents the reciprocal-lattice vectors a*, b* and c* at a gonio­meter angle of ω = χ = φ = 0°. The reciprocal-lattice coordinates Q*′ of each peak at a goniometer angle of ω = χ = φ = 0° are calculated as 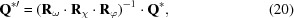
where **R** *_ω_*, **R** *_χ_* and **R** *_φ_* are the 3 × 3 rotation matrices at goniometer angles ω, χ and φ, respectively:
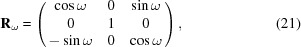


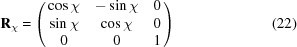
and 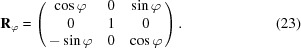

Q* represents the reciprocal-lattice coordinates of each peak determined in the FindPeaks component. An FFT-based indexing algorithm (Steller *et al.*, 1997 ▸) is implemented in the FindCell component. This algorithm is also implemented in the *MOSFLM* X-ray data-processing software (Powell *et al.*, 2013 ▸). The longest unit-cell value is required in the calculation. In most cases, the value for the unit-cell dimension can be used because it is determined beforehand by collecting X-ray diffraction data. If a lattice type is complex (*C*, *I*, *F* or *H*), users must transform the unit cell to a primitive lattice and calculate the longest unit-cell value. For example, if the unit-cell values for a crystal in space group *I*222 are *a* = 93.8, *b* = 99.4, *c* = 102.9 Å, the unit-cell value of the primitive lattice is *a* = *b* = *c* = 85.5 Å. Thus, the longest unit-cell value is 85.5 Å. After the calculation, the **UB** matrix for the primitive lattice is output and used in the next step.

#### 2.2.4. IndexPeaks   

This component calculates the Miller indices of each peak from the **UB** matrix, the reciprocal-lattice coordinates of each peak and the goniometer angle. The Miller indices **h** of each peak are
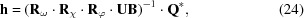
where **R**_ω_, **R**_χ_ and **R**_φ_ are the same as in (21)–(23) [Disp-formula fd22] [Disp-formula fd23]. **UB** is
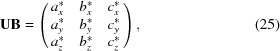
where 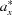 is the projection of the reciprocal-lattice vector a* onto the *x* axis and Q* represents the reciprocal-lattice coordinates of each peak determined in the FindPeaks component. If all of the absolute values of the differences obtained by rounding the calculated Miller indices, and the calculated Miller indices themselves, are less than the threshold, then these peaks are indexed. Protein crystals have a lower crystallinity than organic or inorganic crystals. Because neutrons irradiate the whole crystals and the beam intensity is weaker than that of an X-ray beam, the intensity distribution of the reflections is broader and the accuracy of the peak positions determined by the FindPeaks component could be lower than that for X-ray diffraction data. The default thresholds of *h*, *k* and *l* are set to 0.2, aiming to capture an indexed peak rate of greater than 80%. The *MOSFLM* software uses 0.3 as the default threshold for *h*, *k* and *l* (Powell *et al.*, 2013 ▸). The detector parameters (the distances between each detector face center and the sample, the detector position angles and the detector mis-setting angles), the flight-path length from the CM to the sample and the three-axis goniometer offset angles ω and χ were accurately calibrated by the beamline staff carrying out a least-squares minimization of the summation of the square of the distances between observed peak positions and calculated peak positions in reciprocal space using diffraction data from a single crystal with well known cell dimensions. We can index most peaks using the **UB** matrix determined from all detector peaks at a single crystal orientation. In addition, the indexed peak rate against all observed peaks is calculated to check the accuracy of the **UB** matrix.

#### 2.2.5. ReducedCell   

This component is used for complex lattice types. The candidates for a 3 × 3 transformation matrix from the primitive lattice to the complex lattice are calculated based on the conditions of the reduced cell (de Wolff, 2005 ▸). Unit cells after transformation are also calculated and are used to select a proper transformation matrix from multiple candidates.

#### 2.2.6. LsUBMat   

This component refines the **UB** matrix using indexed peaks. A **UB** matrix determined in the FindCell component is used as an initial value. When the lattice type is complex, the unit cells are transformed and a new **UB** matrix is calculated using the transformation matrix determined in the ReducedCell component. The new **UB** matrix **UB**_N_ is calculated as 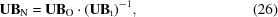
where **UB**_O_ is the **UB** matrix determined by the FindCell component and **UB**_t_ is the transformation matrix determined by the ReducedCell component.

A least-squares minimization is carried out to reduce the summation of the squares of the distances from the observed peak positions determined in the FindPeaks component to the peak positions predicted using the refined **UB** matrix on the detector. Initially, the reciprocal-lattice coordinate Q* is calculated from the Miller indices **h** of the indexed peak, the refined **UB** matrix and the goniometer angles ω, χ and φ, 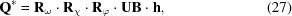
where **R**_ω_, **R**_χ_ and **R**_φ_ are the same as in (23)–(25). The predicted peak positions on the detectors are calculated by the reverse procedure of (12)–(18). λ is calculated from Bragg’s law, 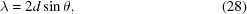
where the lattice distance *d* and scattering angle θ are
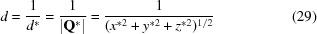
and
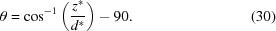
The distance ∊ of each peak is
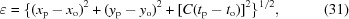
where *x*_o_, *y*_o_ and *t*_o_ are the observed peak position and *x*_p_, *y*_p_ and *t*_p_ are the predicted peak positions. Because the units of the detector position *x*, *y* (cm) and TOF (µs) are different, a scale factor *C* with a default value of 10^−4^ is introduced. The accuracy of the refined **UB** matrix can be evaluated by the differences in Miller indices between the observed and calculated values. The difference ∊_*hkl*_ is defined as 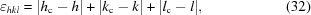
where *h*_c_, *k*_c_ and* l*_c_ are the Miller indices calculated using (24) and *h*, *k* and *l* are the Miller indices of the observed peaks. The more accurate the determined **UB** matrix is, the smaller the ∊*_hkl_* of each peak. As the detector parameters and goniometer offset angles ω and χ were calibrated accurately, the refinement of nine parameters (unit-cell values and crystal orientation) at each crystal orientation can provide an accurate **UB** matrix. If the ∊*_hkl_* of most peaks is less than 0.1, the **UB** matrix is considered to be accurate. In many cases, the deviations of the unrestrained α, β and γ of a unit cell from the α, β and γ determined using X-rays are within ±0.1°.

#### 2.2.7. PeakIntegration   

**Figure 7:**
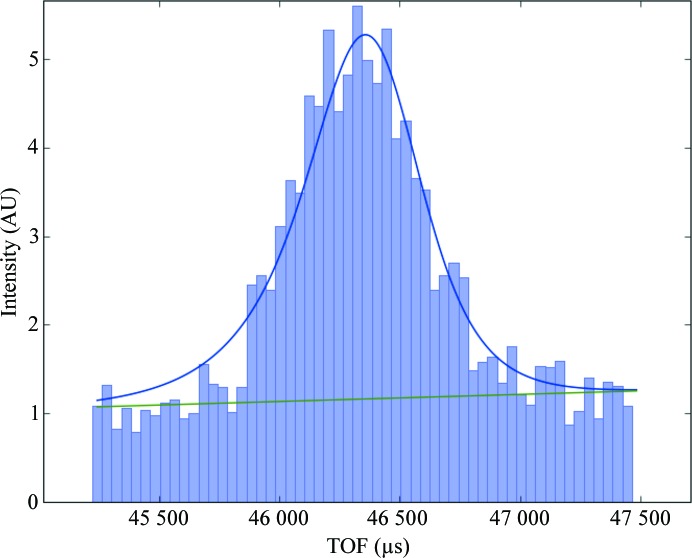
Profile-fitting results for one reflection. Users can confirm the fitting results by viewing the graphic files. Blue solid line, fitting function. Green solid line, background function. Bar graph, one-dimensional intensity distribution in the direction of the TOF axis.

This component predicts the peak positions on detectors and integrates reflection intensities. The intensities and their errors with Lorentz factor correction are calculated. The peak positions on each detector are predicted as follows. The ranges of Miller indices are calculated from the four corner coordinates of the detector faces, TOF range and refined **UB** matrix by using (11)–(19) and (24). The resolution, detector coordinate and TOF are calculated for peaks that have Miller indices within the calculated ranges and do not follow the lattice-type extinction rule. Peaks corresponding to the user-specified resolution range, detector coordinate range and TOF range are selected from these. Peak positions on the detector and the TOF are calculated by the reverse procedure of (11)–(18) and (27)–(30) by considering the TOF offset. By using the visualization component (see §[Sec sec2.3]2.3), users can determine and confirm integration regions before and after peak integration. Because the reflection width in the direction of the TOF axis differs according to the scattering angle, it is recommended to group detectors according to scattering angle and to determine the integration regions separately. If an integration region includes the end of a detector, these reflections are removed from the integration target. Unlike the oscillation method that is used with synchrotron X-rays, only ‘full’ reflections are integrated and it is not necessary to calculate the partialities of each reflection in the TOF method. The summation-integration method or profile-fitting method (Yano *et al.*, 2016 ▸) can be selected as an integration algorithm. Rectangles or elliptic cylinders can be selected as the integration region. When the integration region is an elliptic cylinder, the reflection overlaps are automatically determined from the integration and background regions of the target reflection and the integration regions of the neighboring reflections. Reflections that are judged to be overlapped are removed from the integration target. The profile-fitting method was developed for protein diffraction data and it has been confirmed that the coincidence of the equivalent reflection-intensities index *R*_merge_ and the data-quality indices *R*_p.i.m._, *R*_work_ and *R*_free_ are improved in higher resolution shells. Users can confirm the one-dimensional intensity distributions in the direction of the TOF axis and the fitting results and background functions of each reflection by viewing graphic files (Fig. 7 ▸).

#### 2.2.8. EvaluateConv   

This component merges equivalent reflections and calculates the averaged intensities and their error. In X-ray data-processing software, scaling is carried out to correct various factors after peak integration (Kabsch, 2010 ▸). Because the neutron beam used at iBIX irradiates the whole crystal at all crystal orientations and does not induce radiation damage in protein crystals (O’Dell *et al.*, 2016 ▸), only one crystal is utilized to collect neutron diffraction data. Corrections for variations in the irradiated crystal volume, radiation damage and differences in crystal size and crystalline order are not needed. Corrections corresponding to changes in the intensity beam and variations in detection efficiency have already been performed by the EventToHist component. The differences in detection efficiency between the 30 detectors are corrected by using the total neutron count of the correction data at each detector in this component. The introduction of absorption corrections for incident and diffracted beams is under consideration. After scaling, X-ray data-processing software carries out post-refinement to calculate the partiality of each reflection and accurate unit-cell values. However, in the TOF method all integrated reflections are fully recorded reflections. After neutron data collection, X-ray diffraction data are collected from the crystal irradiated by neutrons or a crystal from a similar crystallization condition as the crystal used for neutron measurements for joint refinement of X-ray and neutron data. The unit-cell value and space group can be determined accurately using the X-ray data. Thus, post-refinement is not carried out in this component.

The unit-cell value and space group are required to calculate the data statistics, and reflections relevant to the space-group extinction rule are removed from the data-statistics calculation. The *hkl* intensity data can be output in the *SCALEPACK* (Otwinowski & Minor, 1997 ▸), *SHELX* (Sheldrick, 2008 ▸) and *GSAS* (Larson & Von Dreele, 2004 ▸) formats. Data statistics, such as the number of observed reflections, the number of independent reflections, the average multiplicity, the completeness (%), *R*_merge_, *R*_p.i.m._ and *I*/σ(*I*) are calculated in terms of resolution, wavelength, scattering angle 2θ, detector and crystal orientation. Graphic files of the data statistics are output to confirm the results visually. The intensity plot, which is similar to a Wilson plot and has a horizontal axis of (sinθ/λ)^2^ and a vertical axis that is the natural logarithm of the averaged intensity of the resolution intervals, is also output as the index with which to determine data quality. After the calculation, the *SCALEPACK* format intensity file can be used for joint refinement of X-ray and neutron data using *PHENIX* (Adams *et al.*, 2010 ▸).

### 2.3. Data-visualization component   

**Figure 8:**
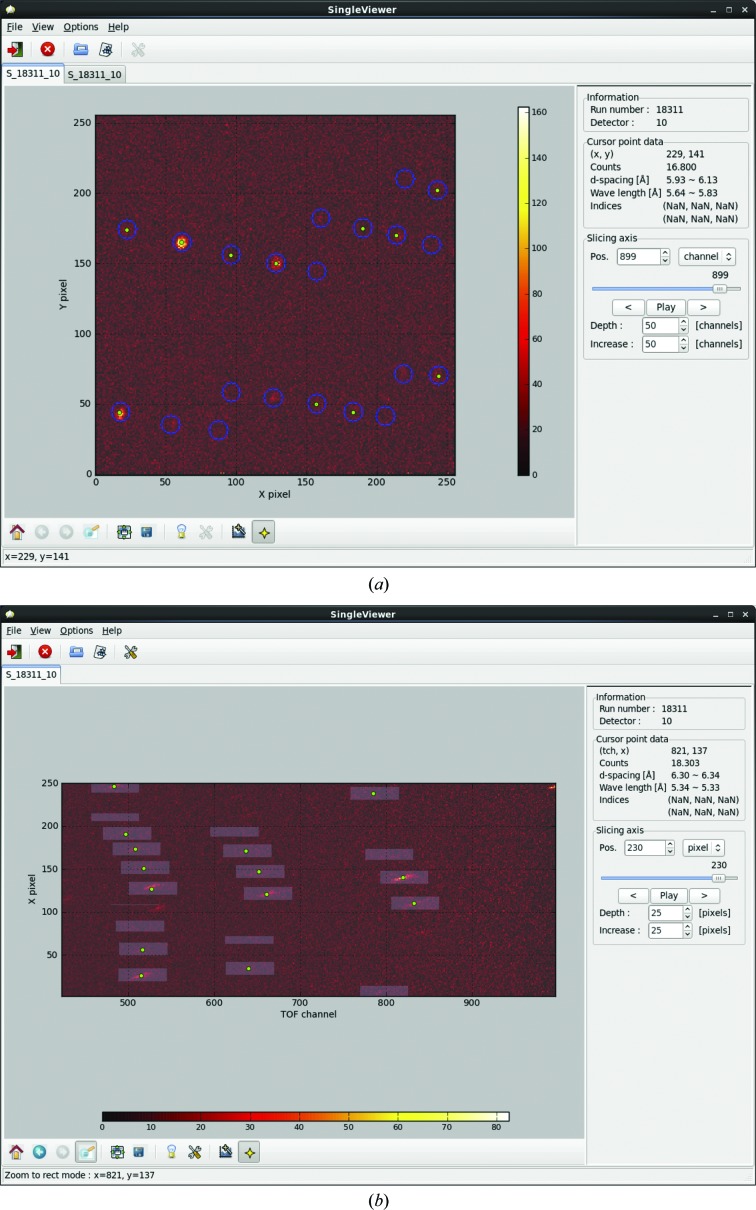
Data-visualization component, showing histogram data with predicted peaks and integrated regions. (*a*) *X*–*Y* slice map. Green points and blue circles represent predicted peak positions and integrated regions, respectively. (*b*) *X*–TOF slice map. Green circles and white rectangles represent predicted peak positions and integrated regions, respectively.

This component is used to visualize the histogram data with searched or predicted peak positions, and to determine and confirm the integrated regions (Fig. 8 ▸). Histogram data record the number of neutron counts at the *x*, *y* and TOF coordinates. Users can check the histogram data as two-dimensional *X*–*Y*, *X*–TOF or *Y*–TOF slice maps and as a one-dimensional TOF profile. The resolution, wavelength, position on the detector and neutron count of each reflection can be displayed using the moving cursor.

## 3. Discussion   

iBIX has been available for user experiments since the end of 2008. To date, neutron diffraction data from many organic, inorganic and protein single crystals have been measured and reaction mechanisms have been proposed (Yokoyama *et al.*, 2012 ▸, 2015 ▸; Ogo *et al.*, 2013 ▸; Unno *et al.*, 2015 ▸; Nakamura *et al.*, 2015 ▸). Thus, it appears that *STARGazer* works well as TOF NPC diffraction data-processing software.

However, the *R*_merge_ of protein diffraction data obtained using TOF diffractometers is not good: for example, the *R*_merge_ values for the overall resolution obtained using synchrotron X-rays, monochromated neutrons (BioDiff, BIX-3 or BIX-4) and TOF neutrons (iBIX or MaNDi or PCS) are approximately 5, 10 and 20%, respectively (Yokoyama *et al.*, 2015 ▸; Chatake *et al.*, 2004 ▸; Yonezawa *et al.*, 2017 ▸; Fisher *et al.*, 2012 ▸; Chen *et al.*, 2011 ▸; Vandavasi *et al.*, 2016 ▸; Langan *et al.*, 2016 ▸). The following four factors can be considered to be the main reasons that the *R*_merge_ of synchrotron X-ray data is lower than that of TOF neutron data. Firstly, the synchrotron X-ray beam intensity is significantly larger than the neutron beam intensity. For example, when comparing the neutron flux of iBIX with the accelerator power of J-PARC (1 MW; Kusaka *et al.*, 2013 ▸) and the photon flux of BL32XU at SPring-8 (Hirata *et al.*, 2013 ▸), the X-ray flux is approximately 10^11^ times larger than the neutron flux. The X-ray beam can provide a higher signal-to-noise ratio and more accurate integration intensity. The measurement time of the neutron diffraction data is clearly insufficient compared with the beam intensity. Secondly, because detectors do not need time resolution for X-rays, ‘integral’-type detectors (image plates or charge-coupled devices) with ∼80% sensitivity can be used. Although the neutron monochromatic method can also use ‘integral’-type detectors (image plates), the TOF method requires ‘differential’-type detectors (scintillator or gas-proportional) with time resolution and ∼40% sensitivity (Niimura & Podjarny, 2011 ▸). The sensitivity to neutrons of ‘differential’-type detectors is lower than that of the ‘integral’ type. Thirdly, the TOF method uses neutrons with continuous wavelengths and the diffraction data are collected by multiple detectors. Equivalent reflections can be measured using different neutron wavelengths and different detectors. The longer the neutron wavelength, the stronger the peak integration intensities of the equivalent reflections before the Lorentz factor correction. The measurement accuracy is different for equivalent reflections and this is an inevitable problem in the TOF method. Fourthly, there are more corrected items (for example, the difference in neutron beam intensities and detection efficiency by wavelength and the differences in detection efficiency between detectors) in the TOF method than in the monochromatic method. There is a possibility that the corrections for the TOF diffraction data are not sufficiently carried out.

*R*_merge_ is an index that shows the degree of coincidence of equivalent reflection intensities and is not suitable as a data-quality index (Karplus & Diederichs, 2012 ▸, 2015 ▸). Even if *R*_merge_ is higher, the *I*/σ(*I*) of the intensity data and the *R*_work_ and *R*_free_ of the refinement can be improved (Weiss, 2001 ▸) and the peak height level of the Bijvoet difference map can be increased by increasing the multiplicity (Suga *et al.*, 2011 ▸). Merged intensity data quality should be evaluated by the correlation between the intensity data and the refined model (*R*_work_ and *R*_free_) and how H atoms and protons of interest are observed in the calculated map. Because the J-PARC accelerator power will be increasing gradually, we will also increase the multiplicity of the diffraction data to improve the data quality.

## 4. Future plans   

In the future, the accelerator power of J-PARC will be increased to a maximum of 1 MW. We will be able to collect diffraction data from crystals with larger unit cells. We will continue to develop *STARGazer* to make it easier to use and will obtain more accurate intensity data. Examples of this include the automation of data processing and the modification of the PeakIntegration component to implement a peak-deconvolution procedure for overlapped peaks from crystals with larger unit cells.
